# Knowledge of orthodontic tooth movement through the maxillary sinus: a systematic review

**DOI:** 10.1186/s12903-018-0551-1

**Published:** 2018-05-23

**Authors:** Wentian Sun, Kai Xia, Xinqi Huang, Xiao Cen, Qing Liu, Jun Liu

**Affiliations:** 1 State Key Laboratory of Oral Diseases, National Clinical Research Center for Oral Diseases, Department of Orthodontics, West China Hospital of Stomatology, Sichuan University, Chengdu, 610041 China; 2 State Key Laboratory of Oral Diseases, National Clinical Research Center for Oral Diseases, Department of Oral and Maxillofacial Surgery, West China Hospital of Stomatology, Sichuan University, Chengdu, 610041 China

**Keywords:** Intrusion, Maxillary sinus, Orthodontics, Root resorption, Systematic review, Tooth movement

## Abstract

**Background:**

To investigate the feasibility, safety and stability of current interventions for moving teeth through the maxillary sinus (MTTMS) by performing a systematic review of the literature.

**Methods:**

The electronic databases PubMed, Embase, CENTRAL, Web of Science, CBM, CNKI and SIGLE were searched without a language restriction. The primary outcomes were parameters related to orthodontic treatment, including orthodontic protocols, magnitude of forces, type of tooth movement, duration and rate of tooth movement, and remolding of alveolar bone and the maxillary sinus floor. The secondary outcomes were safety and stability, including root resorption, perforation of the sinus floor, loss of pulp vitality and periodontal health and relapse.

**Results:**

Nine case reports with 25 teeth were included and systematically analyzed. Fifty to two hundred g of force was applied to move teeth through the maxillary sinus. Bodily movement was accomplished, but initial tipping was observed in 7 cases. The rate was 0.6–0.7 mm/month for molar intrusion and 0.16–1.17 and 0.05–0.16 mm/month for mesial-distal movement of premolars and molars, respectively. Bone formation and remolding of the sinus floor occurred in 7 cases. Root resorption within 6 to 30 months was observed in 3 cases, while no cases of perforation of the sinus floor, loss of pulp vitality, periodontal health impairment or relapse were reported.

**Conclusions:**

At the present stage, no evidence-based protocol could be recommended to guide MTTMS. The empirical application of constant and light to moderate forces (by TAD, segment and multibrackets) to slowly move teeth through or into the maxillary sinus in adults appears to be practical and secure. Bodily movement was accomplished, but teeth appear to be easily tipped initially, potentially resulting in root resorption. However, this conclusion should be interpreted with caution as the currently available evidence is based on only a few case reports or case series and longitudinal or controlled studies are lacking in this area.

## Background

With the development of digitalization and material science in the past few decades, substantial progress has been achieved in orthodontic techniques for more efficient, precise, invisible, comfortable and rapid treatment [1–3]. However, orthodontists commonly encounter predicaments related to dental status, periodontal status, general health, orthodontic technique, anchorage, and other factors that may limit orthodontic treatment [4]. Among these challenges, movement of teeth against anatomic structures, such as the maxillary sinus (MS), appears to be non-evidence-based.

The MS is the largest paranasal sinus, located in the posterior maxilla, and has a close relationship with adjacent structures. It sprouts late in fetal life, existing at birth with a dimension of approximately 3*6*8 mm, and ends its growth in a pyramid shape in adults [5–7]. The MS floor (MSF) consists of a thin bony plate covered with a layer of mucosa. With pneumatization and aging, the floor extends into the posterior alveolar process and forms the alveolar recess, creating protrusions of root apices into the sinus [5]. Generally, the MSF is at the level of nasal floor at puberty and reaches its lowest point with the eruption of the third molars [5, 6]. Morphologically, the sinus-root relationship (SRR) can fall into 5 categories: 0, the root is not in contact with the sinus floor; 1, the MSF curves inferiorly with the root in contact with the MSF; 2, the MSF curves inferiorly with the root projecting laterally on the sinus cavity but its apex is outside the sinus; 3, the MSF curves inferiorly with the root apex projecting on the sinus cavity; and 4, the MSF curves superiorly with part or all of the tooth root enveloped [8]. For individuals with excessive pneumatization, a type-1, 2, 3 or 4 relationship can be common.

The classic theory of orthodontic tooth movement stresses the dynamic balance of bone resorption on the pressure side and deposition on the tension side of the periodontal ligament (PDL) [9]. This theory has been successfully applied by orthodontists to move teeth in the alveolar bone. However, applying this concept to the MS, with potential tooth movement against cortex or soft tissue [10], is more difficult. Consequently, clinicians often fear the uncertainty of moving teeth through the maxillary sinus (MTTMS). However, recent experiments have revealed a particular biomechanical pattern regarding MTTMS. Mechanical stress could induce osteogenesis on the sinus side before bone resorption occurred on the PDL side, and the bone thickness of the sinus wall could be maintained [11–13], potentially indicating the feasibility of MTTMS. Furthermore, concurrent root resorption and higher efficiency of light forces were also observed in these experiments [11–13].

In orthodontic clinics, clinicians may encounter MTTMS. In some cases, planning the distalization of molars or the maxillary dentition to correct type-II occlusion or to achieve a better anterior profile is preferred because this technique has the benefit of avoiding extraction and is reported to be one of the advantages of clear aligners [1, 9, 14]. In other cases, when closure of posterior spaces [15–17], tooth intrusion to create spaces for opposing prosthetics [18, 19] or an alternative non-surgical sinus lift for implant sites [20–22] is required, orthodontists must implement MTTMS. MTTMS determines the feasibility, duration and quality of comprehensive treatment. No systematic reviews on MTTMS are currently available. The purpose of this research was to systematically review the literature and investigate the feasibility, safety and stability of current interventions for MTTMS.

## Methods

This systematic review was conducted generally following the Preferred Reporting Items for Systematic Reviews and Meta-Analysis (PRISMA) checklist [23]. The literature search, data extraction and quality assessment were all performed independently by two reviewers. Any disagreement was resolved by discussion or by consultation with a third party.

### Inclusion criteria

We set the following inclusion criteria to identify eligible studies: (1) Patients with at least one target tooth, defined as a tooth with at least one root protruding into the MS, were investigated. The morphological SRR (type-2, 3 or 4 for distal-mesial movement and type-1, 2, 3 or 4 for intrusion) must be confirmed by radiological diagnosis: periapical films, panoramics or cone-beam computed tomography (CBCT); (2) An orthodontic treatment to move target teeth through the MS was executed; (3) The primary outcomes were parameters related to orthodontic treatment, including orthodontic protocols, magnitude of orthodontic forces, tooth movement type, duration and rate of tooth movement, and remolding of the alveolar bone and MSF. The secondary outcomes were safety and stability, including orthodontically induced root resorption (OIRR), perforation of the sinus floor (Perforation of the sinus floor should be verified by occurrence of sinusitis or by radiological findings in sinus. The integrity of lamina dura should be assessed by radiography: periapical films, panoramics or CBCT, while the sinus membrane should be assessed with CT/CBCT, MRI or endoscopy [5, 18, 19, 24–28].), pulp vitality loss, and periodontal health impairment and relapse; and (4) The study was a clinical study, including randomized clinical trial, controlled clinical trial, cohort study, case-control study, cross-sectional study and case report.

### Search strategy

Online searches were conducted in electronic databases, including PubMed, Embase, CENTRAL, Web of Science, Chinese Biomedical Literature Database (CBM), China National Knowledge Infrastructure (CNKI), without restriction of language. Grey literature was searched in the System for Information on Grey Literature in Europe (SIGLE). We used MeSH terms as well as free text words, and the key words were “maxillary sinus,” “orthodontics,” “orthodontic*,” “tooth moving,” and “tooth movement” for all databases. The reference lists of relevant articles were manually searched for additional studies. The searches were initially conducted in January 2017 and were updated on May 16, 2017.The search strategies are presented in Table 1.

**Table 1:** Search strategies for all databases (updated on May 16, 2017)

steps	PubMed	Embase	CENTRAL	Web of science	CNKI	CBM	SIGLE
1	“Maxillary Sinus” [Mesh] (9226)	Maxillary Sinus.mp. or Maxillary sinus/ (14263)	Maxillary Sinus.mp. or Maxillary Sinus/ (424)	Maxillary Sinus (21210)	Maxillary Sinus (8251)	“Maxillary Sinus” [Mesh] (3483)	Maxillary Sinus (26)
2	Maxillary Sinus (15823)	Orthodontics.mp. or Orthodontics/ (34932)	Orthodontics.mp. or Orthodontics/ (636)	Orthodontics (29732)	Orthodontics (12742)	Maxillary Sinus (7208)	Orthodontics (85)
3	“Orthodontics” [Mesh] (48362)	Orthodontic*.mp. (51672)	Orthodontic*.mp. (2402)	Orthodontic* (67283)	Tooth movement (1045)	“Orthodontics, Corrective” [Mesh] (11645)	Orthodontic* (235)
4	Orthodontics (63379)	Tooth moving.mp. (12)	Tooth movement.mp. or Tooth Movement/ (382)	Tooth moving (54506)	2 OR 3 (15616)	Orthodontics (16725)	Tooth movement (14)
5	Orthodontic* (62688)	Tooth movement.mp. (2970)	2 OR 3 OR 4 (2423)	Tooth movement (59130)	1 AND 4 (23)	“Tooth movement” [Mesh] (1104)	Tooth moving (3)
6	“Tooth Movement Techniques” [Mesh] (7834)	2 OR 3 OR 4 OR 5 (52143)	1 AND 5 (1)	2 OR 3 OR 4 OR 5 (157498)		Tooth movement (2280)	2 OR 3 OR 4 OR 5 (245)
7	Tooth movement (10981)	1 AND 6 (125)		1 AND 6 (305)		1 OR 2 (7208)	1 AND 6 (0)
8	Tooth moving (327)					3 OR 4 OR 5 OR 6 (18312)	
9	1 OR 2 (15823)					7 AND 8 (28)	
10	3 OR 4 OR 5 OR 6 OR 7 OR 8 (71532)						
11	9 AND 10 (195)						

*CENTRAL* Cochrane Central Register of Controlled Trials, *CNKI* China National Knowledge Infrastructure, *CBM* Chinese Biomedical Literature Database, *SIGLE* System for Information on Grey Literature in Europe

### Data extraction and analysis

Information regarding the characteristics and outcomes of the included studies was extracted. Specifically, the following characteristics were identified: country, age, sex, sample size, target teeth, SRR, source of active force and radiological method. The outcomes were those items defined in the inclusion criteria above.

## Results

### Characteristics of the included studies

**Fig. 1:**
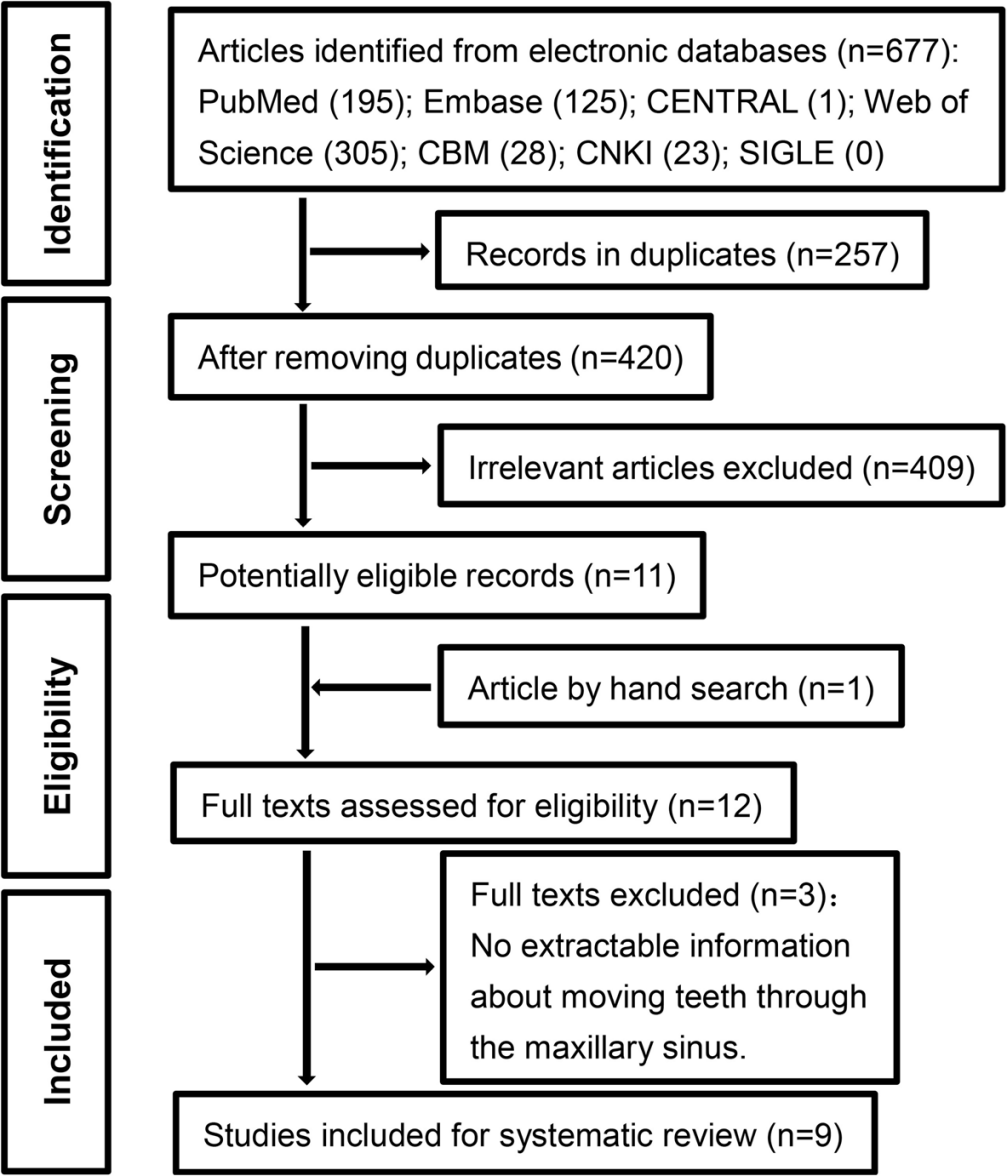
PRISMA flow diagram showing the search and selection process

The online search yielded 677 articles. After excluding duplicate and irrelevant articles by reading titles and abstracts, 11 full texts remained. Then, the reference lists of these articles were read and one additional study was identified. No prospective or retrospective controlled clinical studies were found. Nine case reports meeting the inclusion criteria were included and systematically analyzed (Fig. 1). The characteristics of the 9 case reports are presented in Table 2.

**Table 2:** Characteristics of included case reports

Author(s)	Country	Age	Sex	Teeth	SRR	Mechanics	Active force system used	Radiographic view	Conclusion
Cacciafesta (2001) [17]	Denmark	25	F	2(27,28)	type-3	segment; multibracket	coil spring	panoramic; periapical film	Teeth can be moved into anatomical sites lacking periodontium provided that the orthodontist uses an appliance that generates both constant forces and constant moment to force ratios.
Re (2001) [22]	Italy	24	F	1(25)	type-4	segment	T-loop	panoramic; periapical film	The clinical findings of this study indicate that with a proper orthodontic force system, a tooth can be displaced through the sinus area, and the sinus lift surgical augmentation procedure can be avoided.
Yao (2004) [19]	Taiwan	31	F	2(26,27)	type-3	segment; miniplate, miniscrew	elastic power chain	panoramic; periapical film 3D digitizer	The biological responses of teeth and the surrounding bony structures to intrusion appeared normal and acceptable. Furthermore, the periodontal health and vitality of the teeth were sufficiently maintained even after a 1-year follow-up.
Kravitz (2006) [18]	USA	44	F	1(16)	type-3	miniscrew	elastic power chain; coil spring	panoramic	A supraerupted maxillary molar can be successfully intruded within the maxillary sinus cortical floor using two orthodontic miniscrews. Short-term molar intrusion can be achieved without clinically detectable apical root resorption.
Oh (2014) [16]	USA	41	F	4(15,17,25,28)	type-2:15,25,28 type-4:17	multibracket	elastic power chain; coil spring; tip-back bend; double helical loop	panoramic; periapical film; CBCT	Successful tooth movement through the maxillary sinus can be achieved without noticeable side effects. New bone formation along the course of tooth movement and changes in the size and shape of the maxillary sinus were observed. Maintaining light continuous forces and moving teeth at a slow rate were key in accomplishing bodily movement and direct bone resorption.
Park (2014) [15]	Japan	31	M	4(14,16,24,26)	type-2:14,24 type-3:16,26	multibracket; TAD	T-loop; Intrusion archwires; TAD	panoramic; CBCT	Spaces from tooth extractions can be closed by bodily movement through anatomic barriers such as the maxillary sinus, but in view of the proximity of the maxillary sinus floor and maxillary root tips, orthodontists must be particularly cautious when doing this.
Saglam (2014) [21]	Turkey	54	M	1(25)	type-3	multibracket	coil spring	panoramic; periapical film;	Modification of the sinus floor by orthodontic treatment may be an alternative treatment strategy for patients requiring a sinus lifting procedure due to pneumatization of maxillary sinus.
Carvalho (2014) [20]	Brazil	38	M	1(15)	type-3	multibracket	unknown	periapical film	Orthodontic movement is a safe and predictable procedure and may replace sinus lift and graft procedures for patients who smoke or for individuals with a history of sinusitis. The procedure also allows implant placement in an area of mature bone rather than in grafted bone, which may be a favorable factor for osseointegration.
Kuroda (2016) [14]	Japan	29	F	9(14,15,16,17,23,24,25,26,27)	type-2:14 type-3:15,16,17,23,24,25,26,27	multibracket; TAD	coil spring	panoramic	Interradicular miniscrews are useful for distalizing the maxillary dentition to correct class II malocclusion. With this new strategy, group distalization with miniscrews enables a simpler treatment with greater predictability.

*SRR* sinus-root relationship, *TAD* temporary anchorage device, *CBCT* cone beam computed tomography

### Results of quality assessment

All included studies were case reports and consequently had a relatively high risk of bias.

### Study outcomes analysis

In total, nine adult subjects with 25 target teeth were included. All target teeth had a type-2, 3 or 4 SRR (Table 3).

**Table 3:** Outcomes of the 9 included case reports

Author(s)	SRR	Force magnitude	Moving distance through sinus	Duration	Tooth movement type	Bone forming and remodeling of the sinus floor	Side effects	Follow-up and relapse
Cacciafesta (2001) [17]	type-3	50 g	4–5 mm (half the width of a molar)	unknown	bodily mesially; up-righting	Bone formation took place.	minimal root blunting; No marginal bone loss was visible.	unknown
Re (2001) [22]	type-4	50 g/mm	6 mm	6 months	bodily distally (tipping, translation, root movement)	Alveolar bone formation and direct remodeling of the sinus lamina dura occurred.	Pulp vitality, bone support and normal width of the periodontal ligament were maintained.	unknown
Yao (2004) [19]	type-3	150–200 g	3 mm	5 months	intrusion, slight distal tipping	The lamina dura followed molar intrusion and bone remodeling was achieved.	Periodontal health and vitality of the teeth were maintained.	1 year; Periodontal health and vitality of the teeth were well maintained.
Kravitz (2006) [18]	type-3	100**–**150 g	4.4 mm	6 months	intrusion, palatal crown tipping	Radiograph showed intact lamina dura around the first molar within the floor.	no radiographically evident root resorption.	unknown
Oh (2014) [16]	type-2:15,25,28 type-4:17	light forces	25: 6 mm; 28: 10 mm; 10 mm (15–17)	70 months	25: bodily distally (tipping, up-righting); 28: bodily mesially (tipping, up-righting); 15: bodily distally; 17: bodily mesially	Signs of sinus wall modeling and new alveolar bone deposition were observed in the direction of tooth movement.	No apparent root resorption was observed, and the alveolar bone level was maintained.	18 months; Occlusion and normal overjet and overbite were maintained.
Park (2014) [15]	type-2:14,24 type-3:16,26	light forces	14–16: (bodily 2–3 mm, up-righting 15–20°) 24–26: (bodily 1–2 mm, up-righting 20–25°)	30 months	14, 24: bodily distally, up-righting; 16, 26: bodily mesially, rotated mesially, up-righting	The floor of the sinus did not displace coronally during orthodontic approximation of these teeth.	Some areas showed signs of apical root resorption.	1 year; Stable occlusion and the orthodontic treatment results were maintained.
Saglam (2014) [21]	type-3	unknown	7 mm	unknown	bodily distally	Alveolar bone formation and remodeling of the sinus floor occurred.	Maintained pulp vitality and bone support without loss of the connective tissue attachment.	2 years; Acceptable intraoral tissue health was observed after 2 years.
Carvalho (2014) [20]	type-3	mild and moderate	7 mm	6 months	bodily distally	The cortical bone and sinus mucosa displaced the maxillary sinus floor during bone and periodontal remodeling.	Radiographically evident root resorption was observed.	unknown
Kuroda (2016) [14]	type-2: 14 type-3:15,16,17,23,24,25,26,27	200 g	4–5 mm	28 months	bodily distally (tipping, up-righting)	unknown	No serious root resorption.	5 years; Occlusion and facial profile were stable.

*SRR* sinus-root relationship, *Moving distance through sinus* the distance by which the tooth was moved through the maxillary sinus

#### Protocol and magnitude of forces

Cacciafesta et al. [17] used segments to protract tooth numbers 27 and 28 mesially, and the force was 50 g by coil spring. Re et al. [22] used an endosseous implant in the retromolar area and a T-loop to move tooth number 25 distally, and the active load was 50 g/mm. In Kravitz et al.’s article [18], tooth number 16 was intruded using temporary anchorage devices (TADs), and the forces were 100 g by elastic power chain in the initial 2 months and 150 g by coil spring in the next 4 months. Yao et al. [19] also used TADs to intrude two adjacent molars (26 and 27), and the forces were 150–200 g by elastic power chain. Kuroda et al. [14] performed group distalization of the maxillary dentition using multibrackets and TADs, and 9 teeth were moved distally through the MS bilaterally with a load of 200 g by coil spring. Oh et al. [16], Park et al. [15], Saglam et al. [21] and Carvalho et al. [20] used multibrackets to move maxillary teeth mesially or distally through the MS (TADs were utilized in Park et al.’s article). In these reports, “light forces” or “mild to moderate forces” were used. Generally, light to moderate forces (50–200 g) were applied to accomplish MTTMS.

#### Tooth movement type

In general, 7 articles reported MTTMS in the sagittal direction [14–17, 20–22], and 2 articles reported molar intrusion into the MS in the vertical direction [18, 19]. For tooth movement in the sagittal direction, all authors reported distal or mesial bodily movement of the target teeth [14–22]. However, Cacciafesta et al. [17], Re et al. [22], Oh et al. [16], Park et al. [15] and Kuroda et al. [14] revealed that the overall translation consisted of processes of initial tipping followed by up-righting. Saglam et al. [21] and Carvalho et al. [20] described distal bodily movement of the second premolars, but no details were provided in their reports. For molar intrusion into the MS, Yao et al. reported intrusion of tooth numbers 26 and 27 with slight distal tipping [19], while Kravitz et al. reported intrusion of tooth number 16 with palatal crown tipping [18].

#### Duration and rate of tooth movement

For tooth movement in the sagittal direction, Re et al. moved tooth number 25 by 6 mm distally in 6 months [22]. Oh et al. reported distal movement of 5 mm for tooth number 25, mesial movement of 10 mm for tooth number 28, and opposing movement of 10 mm between tooth numbers 15 and 17 in 70 months [16]. Opposing movements of 2–3 mm between tooth numbers 14 and 16 and 1–2 mm between tooth numbers 24 and 26 were achieved in 30 months in Park et al.’s article [15]. Carvalho et al. moved tooth number 15 by 7 mm distally in 6 months [20]. In addition, Kuroda et al. achieved group distalization of the maxillary dentition of 4–5 mm in 28 months [14]. For tooth movement in the vertical direction, 3-mm intrusion in 5 months and 4.4-mm intrusion in 6 months for molars were reported by Yao et al. and Kravitz et al., respectively [18, 19]. Overall, for molar intrusion into the MS, the individual cases showed a rate of 0.6–0.7 mm/month, and for distal-mesial movement, rates of 0.16–1.17 and 0.05–0.16 mm/month were reported for premolars and molars, respectively.

#### Alveolar bone formation and remolding of the sinus floor

Re et al. [22], Saglam et al. [21], and Carvalho et al. [20] moved the second premolars distally through the MS with pneumatization into the alveolar bone. Alveolar bone formation occurred in the moving direction, along with direct remolding of the sinus lamina dura and sinus lift, and implants were subsequently inserted in the previous positions of the second premolars. Likewise, alveolar bone formation was observed in the studies of Cacciafesta et al. [17] and Oh et al. [16], and signs of sinus wall modeling were also observed in Oh et al.’s study. In terms of molar intrusion, Yao et al. and Kravitz et al. found that the lamina dura followed the course of molar intrusion, and bone remolding during intrusion was achieved in Yao et al.’s case [18, 19].

#### Safety and side effects

First, radiographically evident OIRR was reported by Cacciafesta et al. [17], Park et al. [15] and Carvalho et al. [20], whereas no apparent OIRR was reported by Kravitz et al. [18], Oh et al. [16] and Kuroda et al. [14]. Second, no perforation of the sinus floor or loss of pulp vitality was reported in the 9 cases [14–22]. Third, standard periodontal control measures were adapted by Cacciafesta et al., Re et al., Oh et al., and Saglam et al. [16, 17, 21, 22], and they reported that bone support and periodontal health were maintained. Similar result was observed by Yao et al. [19].

#### Stability and relapse

Oh et al. [16], Park et al. [15] and Kuroda et al. [14] reported stable occlusion after follow-ups of 1.5, 1 and 5 years, respectively. The periodontal health and vitality of the teeth were maintained in Yao et al.’s case [19]. Saglam et al. reported acceptable intraoral tissue health after 2 years [21].

## Discussion

This systematic review intended to determine the feasibility, safety and stability of current interventions for MTTMS. Nine case reports representing the available human clinical studies were included. In general, the present study indicated the feasibility of MTTMS. However, the difficulty of the moving process varied substantially, possibly indicating the heterogeneity among clinical measures and internal anatomic structures and the inherent bias of case reports.

In MTTMS, bodily movement is desired. The key biomechanical objective is uniform distribution of orthodontic forces along the PDL and the line of the active force passing through the center of resistance [29, 30]. Carefully designed segments or TADs can produce approximate determinate force systems and may facilitate bodily movement [9, 17, 31, 32]. Considering the anatomic variability of the MSF and the complexity of the SRR [8], techniques for better control in three dimensions should be developed, especially for patients with primarily regional complaints. To achieve tooth movement by frontal resorption, mild and constant forces (35–60 g, 70–120 g and 10–20 g for tipping, bodily and intrusion movement, respectively) are recommended. However, considering the amount of resistance in sliding mechanics [9, 30], the decay rate of forces and the root numbers of the posterior teeth, the magnitude of 50–200 g of force applied in the included cases seems safe.

In the present study, most cases showed initial stages of tipping through the MS, which is consistent with a previous study [33]. Deviation from ideal bodily movement may reflect expression of a well-designed pure Newtonian mathematical force system applied on the in vivo PDL. Orthodontic forces are derived from deformation of some parts of existing appliances; however, each appliance has a particular load deflection rate, and the decay rates of the counterparts (i.e., the moment of force and the moment of couple) in the equilibrium system are not equal, and consequently, the moment to force ratio constantly changes, resulting in constant changes in the center of rotation and difficulty in maintaining translation [34]. Moreover, in the MS, the distribution of bone density along the axis of a tooth must be considered. The coronal part of the root is more likely to move against cancellous bone while the apical part is more likely to move against cortical bone [9]. Therefore, the tooth is easily tipped toward the moving direction. Furthermore, for molar intrusion, the accompanying tipping may reflect different resistances among roots [19].

Moving at low speeds is a prerequisite for compensatory bone regeneration [16, 35]. Therefore, applying light and continuous forces is the best strategy to achieve the ideal speed for moving teeth. Particularly, the cask effect regarding the moving rate in MTTMS is probably due to cortical anchorage [9]. In Oh et al.’s case, the location of the roots against the cortical bone of the sinus wall provided nearly absolute anchorage in the first 3.5 years [16]. In such cases, only light forces are appropriate as heavy forces against the cortical bone will reinforce the anchorage and cause additional OIRR. However, individual heterogeneity may exist in this regard because no other authors reported such an extreme situation, and a wide range of rate was reported across cases.

According to the theory of tooth movement, new physiological bone along the course of the moved tooth can be harvested [9, 36]. In the past few decades, the development of implant sites with the help of orthodontic tooth movement has been shown to be practical [36], and in several cases, this technique was successfully applied in the MS area [20–22]. One major concern, however, is maintaining the intact membrane of the sinus floor. First, in surgical sinus augmentation procedures, the floor is mechanically and instantaneously lifted by applying graft materials or alveolar bone blocks [37, 38]. This shows the endurance and reparability of the sinus floor as it may adapt to slow and mild tooth movement. Second, recent studies have revealed that under stressful stimulation, bone deposition on the sinus side preceded resorption on the PDL side, and the amount of bone in the sinus wall was maintained or increased. This mechanism may partially account for bone remolding of the sinus floor [11, 12].

Currently, the etiology of OIRR is unclear. However, comprehensive orthodontic treatment, particularly the application of heavy forces, undoubtedly cause increases in the incidence and severity of root resorption [39]. Moving a tooth against the cortical bone is more likely to cause heavy root resorption, but serious OIRR was not reported in any of the included cases, perhaps because of the light forces applied. However, lack of detection of serious OIRR in some cases may reflect a limitation of radiographic approaches [18, 19, 22]. On the one hand, there is hysteresis between histological and radiographical changes, and early root resorption could only be detected on radiographs after 6–12 months [39]. On the other hand, panoramics or periapical films were used in most cases [14, 17–22], but their diagnostic accuracy may be insufficient [39, 40]. For retention, wrap-around or bonded retainers were mainly used for the maxillary teeth, and implants and subsequent prosthetic crowns were installed adjacent to or opposing the moved teeth. All these factors contributed to good retention and stability after MTTMS.

### Limitations

Currently, the comprehension of MTTMS is limited. First, prospective controlled clinical trials with large samples are not available, so an optimal orthodontic protocol has not been established. Second, the techniques used to evaluate the SRR, OIRR and perforation of the sinus floor have low accuracy [41] and applying panoramics and periapical films can introduce errors in patient selection or outcome measurements. To improve accuracy, CBCT is an alternative strategy [8, 24, 25, 40, 42]. Third, the results of basic research simulating MTTMS are not necessarily authentic. Although the discovery of recent research was novel and was partly consistent with some clinical observations [11–13], the studies involved only a 2-week observation period on mouse models. And the results were not entirely consistent with the long-term findings in a previous biopsy report in human in which osteoclasts and obvious lamina dura resorption were observed on the sinus side [41]. Therefore, more basic studies with consistent models should be conducted to confirm these results. Lastly, during orthodontic tooth movement, some side effects such as severe root resorption, osseous perforation, and sinus perforation may be beyond orthodontists’ control. These cannot be macroscopically or radiologically detected but can be verified histologically. Clinicians should determine accurate diagnoses with consideration of anatomical structures before treatment, execute careful protocols, and conduct progress evaluations throughout treatment [39–41].

## Conclusion

At the present stage, no evidence-based protocol could be recommended to guide MTTMS. The empirical application of constant and light to moderate forces (by TAD, segment and multibrackets) to slowly move teeth through or into the maxillary sinus in adults appears to be practical and secure. Bodily movement could be accomplished, but teeth seem to be easily tipped initially, potentially resulting in root resorption. However, this conclusion should be interpreted with caution as the currently available evidence is based on only a few case reports or case series, and longitudinal or controlled studies are lacking in this area.

## Abbreviations

CBCT: Cone-beam computed tomography
MS: Maxillary sinus
MSF: Maxillary sinus floor
MTTMS: Moving teeth through the maxillary sinus
OIRR: Orthodontically induced root resorption
SRR: Sinus-root relationship
